# A state-level history of opioid overdose deaths in the United States: 1999-2021

**DOI:** 10.1371/journal.pone.0309938

**Published:** 2024-09-06

**Authors:** David Kline, Staci A. Hepler, Noa Krawczyk, Ariadne Rivera-Aguirre, Lance A. Waller, Magdalena Cerdá

**Affiliations:** 1 Division of Public Health Sciences, Department of Biostatistics and Data Science, Wake Forest University School of Medicine, Winston-Salem, North Carolina, United States of America; 2 Department of Statistical Sciences, College of Arts and Sciences, Wake Forest University, Winston-Salem, North Carolina, United States of America; 3 Center for Opioid Epidemiology and Policy, Division of Epidemiology, Department of Population Health, New York University Grossman School of Medicine, New York, New York, United States of America; 4 Department of Biostatistics and Bioinformatics, Rollins School of Public Health, Emory University, Atlanta, Georgia, United States of America; University of Connecticut Health Center: UConn Health, UNITED STATES OF AMERICA

## Abstract

We examined a natural history of opioid overdose deaths from 1999-2021 in the United States to describe state-level spatio-temporal heterogeneity in the waves of the epidemic. We obtained overdose death counts by state from 1999-2021, categorized as involving prescription opioids, heroin, synthetic opioids, or unspecified drugs. We developed a Bayesian multivariate multiple change point model to flexibly estimate the timing and magnitude of state-specific changes in death rates involving each drug type. We found substantial variability around the timing and severity of each wave across states. The first wave of prescription-involved deaths started between 1999 and 2005, the second wave of heroin-involved deaths started between 2010 and 2014, and the third wave of synthetic opioid-involved deaths started between 2014 and 2021. The severity of the second and third waves was greater in states in the eastern half of the country. Our study highlights state-level variation in the timing and severity of the waves of the opioid epidemic by presenting a 23-year natural history of opioid overdose mortality in the United States. While reinforcing the general notion of three waves, we find that states did not uniformly experience the impacts of each wave.

## Introduction

The United States (US) is in the midst of an unprecedented overdose crisis. Since 1999, more than one million Americans have died of an overdose, reaching a record high of more than 106,000 in 2021 [1]. Overdose is now the leading cause of death among Americans 18–45 years old [2], and overdose deaths are increasing in all age groups [3]. In 2021, there were more drug overdose deaths in the US than in any year since records began [1, 4].

The overdose crisis has been described as following three waves [5], characterized first by a rise in overdoses involving prescription opioids, followed by a rise in overdoses involving heroin, and finally characterized by overdoses driven predominately by illegally manufactured synthetic opioids such as fentanyl, which continues to present day [6]. These waves coincided with shifts in the opioid supply, including first a four-fold increase in the volume of dispensed prescription opioids, followed by a rise in the US heroin supply and a drop in heroin price; and finally, the introduction of fentanyl (30–40 times stronger than heroin and one-tenth of heroin’s price by weight) into the unregulated drug market [7–10].

Despite this general trend, the rise in overdoses did not manifest in the same way across the US. In the early 2000s, opioid overdose deaths first experienced a marked rise in largely rural states and in deindustrialized areas in Appalachia, including West Virginia, Kentucky and Ohio [11]. With a shift towards heroin-involved overdoses, the rate of overdose accelerated in the Midwest and Northeast [12]. The most recent rise in overdoses, driven by synthetic opioids, has spread across the country, with a particularly high increase in the East Coast, the Midwest, and most recently, the West Coast [13].

While prior studies have primarily focused on national and regional trends, few studies have characterized state-level variation in the timing and severity of the three waves of the epidemic. In this paper, we present a comprehensive state-level “history” of the opioid epidemic in the United States by estimating death rates by state and drug involvement from 1999–2021 within a single unified model. We apply our model to estimate the timing and magnitude of changes in overdose death rates involving prescription opioids, heroin, synthetic opioids, and unspecified drugs within each state. By doing so within a single model, we provide a holistic view of state-level variation of the opioid overdose epidemic over 23 years and contribute nuance that enhances our understanding of the three waves.

Achieving this aim presents several statistical challenges. First, we need to estimate the timing of multiple unknown changepoints to capture the onset of each wave as well as increases and decreases in rates due to changing use patterns, drug supplies, and policy interventions. Thus, we need to estimate the timing of multiple unknown changepoints within each drug type to detect these changes. Second, rates of overdose death follow complex temporal trajectories that vary considerably by state and are not easily modeled through simple log linear parametric functions. More flexible models, like splines, do not typically enable the detection of change points as abrupt changes are functionally smoothed over. Third, we need to quantify uncertainty about the presence of a changepoint and the magnitude of change in the death rate in a way that is interpretable for all states over 23 years. To overcome these challenges, we develop a multivariate Bayesian hierarchical model that allows us to flexibly estimate the timing and magnitude of multiple changes in overdose trends while accounting for regional structure, borrowing strength to stabilize estimates based on smaller counts, and fully propagating uncertainty.

## Materials and methods

### Data

We obtain overdose death counts from the restricted-use mortality data set produced by the National Center for Health Statistics for deaths occurring from 1999 to 2021 in the continental United States [14]. We include all overdose deaths as indicated by the presence of International Classification of Disease, Tenth Revision (ICD-10) codes for accidental or unintentional drug poisoning (X40-X44), intentional self-poisoning or suicide (X60–64), assault or homicide (X85), or undetermined intent (Y10-Y14) in the death record. Based on ICD-10 multiple cause of death codes, we further classify each death by drug involvement: prescription opioids (T40.2: Other opioids, T40.3: Methadone), heroin (T40.1), synthetic opioids (T40.4), and unspecified drugs or narcotics (T50.9: Other and unspecified drugs, medicaments and biological substances, T40.6: Other and unspecified narcotics but not T40.1, T40.2, T40.3, T40.4). We include unspecified drugs or narcotics as a category to explore potential variation in reporting across space and time. We note that often T40.6 is included in counts of deaths involving any opioid, but since it does not specify any of the three drug types of interest, we include it in the unspecified category. Deaths caused by multiple drugs of interest are counted within each category present in the death record with the exception of the unspecified category which only captures deaths not otherwise counted in one of the three other categories. Thus, the unspecified category counts deaths that would have otherwise been excluded if looking solely at the other three categories. We aggregate the number of deaths attributed to each drug category to the state level by year of death. Defining drug categories in this way provides a sense of the drug environment in a given state and year but counts across categories will not aggregate to the total number of deaths in the state due to counting some deaths in multiple categories. Additional discussion on the classification of drug overdose deaths can be found in Robinson (2017) [15]. To enable the calculation of rates, estimates of each state’s population are obtained from CDC WONDER [16] for each year studied. We identify the region for each state as defined by the Centers for Medicare and Medicaid Services (CMS) (S1 Table) to include as a covariate to account for regional heterogeneity.

### Statistical considerations

The main objective of the analysis is to describe state-level variation in the timing and magnitude of change in overdose death rates involving prescription opioids, heroin, synthetic opioids, and unspecified drugs. To do so, we adapt a multivariate Bayesian multiple changepoint model [17] that allows for an unknown number of changepoints for each state and drug type. Our model provides a flexible framework for estimating each time series, allows for full quantification of uncertainty within a unified model through Bayesian inference, and allows us to account for multivariate dependence across drug types.

First, we explain the intuition for the multiple changepoint model and how it aligns with the epidemiologic questions of interest. Under our model, each state has an overdose death rate for each drug type in 1999. We assume the death rates stay the same unless there is a disturbance to the system that leads to an increase or decrease in the rates. For example, such disturbances could be changes in policy interventions, the local drug supply, use patterns, or the social environment. These disturbances reflect changepoints, or “regime changes” [17], that mark a change in the death rate. If a change is not estimated to have occurred, contiguous time periods are said to be in the same regime because they have the same underlying mean death rate. That is, the observed death count is compatible with expected variation around expected counts based on the existing estimated death rate. When death counts become incompatible with the existing estimated rate, the model infers the presence of a changepoint, which signals a new estimated rate and the start of a new regime. This process is jointly carried out for each drug type in each state and results in very flexible, semi-parametric estimates of the time series of death rates. Our approach allows us to capture the complex ebbs and flows of the waves of the epidemic without imposing restrictive parametric assumptions.

We briefly describe the Bayesian hierarchical model here but leave full details to the S1 Appendix. For each state, year, and drug type, we assume the death counts follow a Poisson distribution with a mean of the total population in that state and year times the current death rate. The death rate is specific to a drug type in a state and the set of contiguous years within the same regime (i.e., no estimated changepoints). The probability of a changepoint within a given state, year, and drug type is modeled using a centered autologistic model [18] that includes a regional random effect and temporal autoregressive structure. The regional effect accounts for potential regional correlation in the timing of changes in the death rates and is multivariate to capture dependence across drug types. The autoregressive structure accounts for a notion of volatility—that states experiencing a change the year prior may be more likely to experience a subsequent change.

Since we are fitting the model within the Bayesian paradigm, we must assign prior distributions and choose proper but diffuse prior distributions for all parameters. To fit the model, we ran a Markov chain Monte Carlo algorithm using nimble [19] in R for 500,000 iterations. We discarded the first 250,000 as burn-in and thinned the remaining samples by 50. Convergence was assessed visually using trace plots. Computation took approximately 5.5 hours using the DEAC cluster [20]. Code is available in S1 File. All maps were generated with the maps R package [21].

## Results

**Fig 1:**
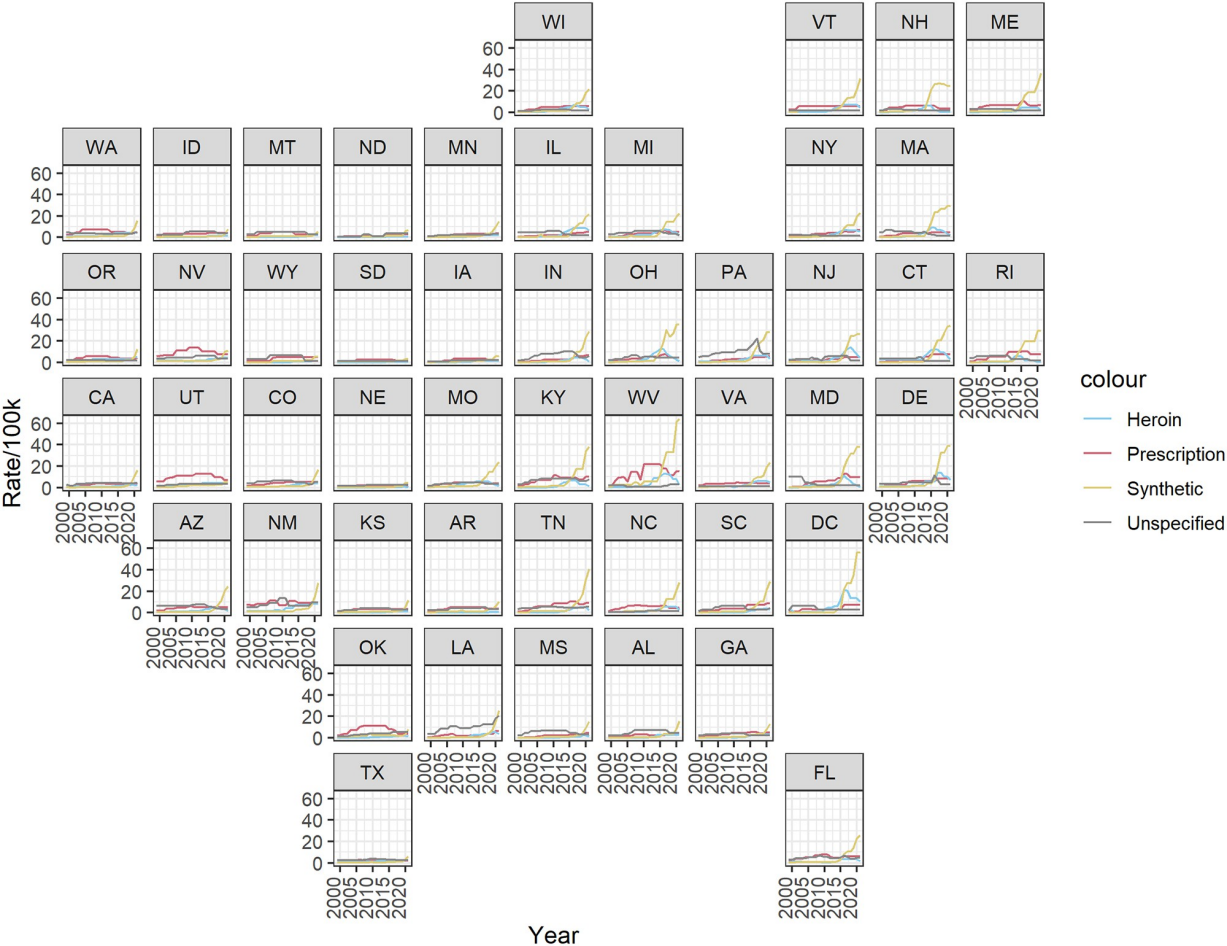
State-level time series geofacet plot. Posterior median estimates of the overdose death rates per 100,000 residents related to each drug type from 1999–2021 for each state.

**Fig 2:**
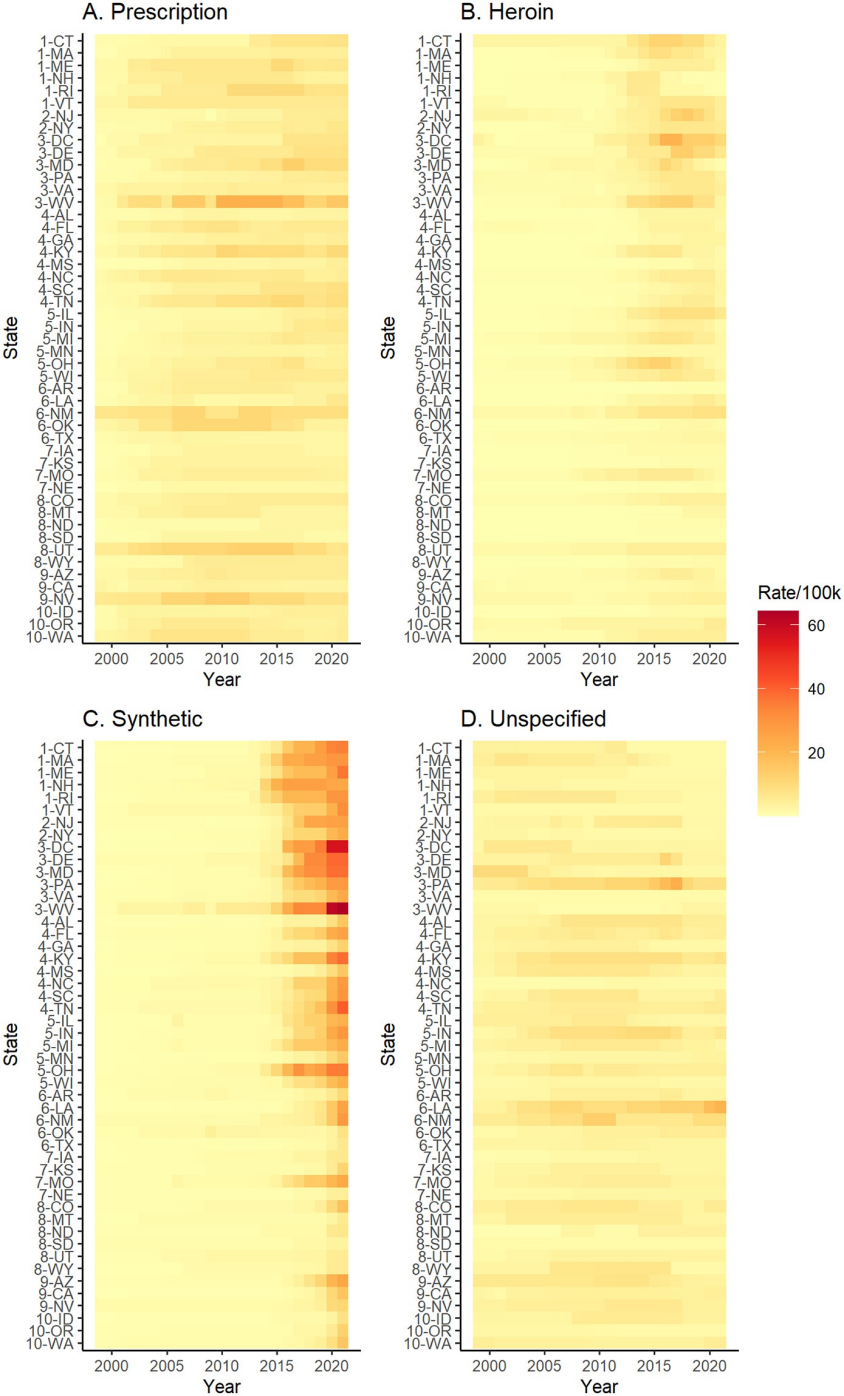
State-level time series heatmaps. Posterior median estimates of the overdose death rates per 100,000 residents related to each drug type from 1999–2021 for each state ordered by Centers for Medicare and Medicaid Services (CMS) region. The number preceding each state abbreviation is the CMS region for that state.

We begin with an overview of the figures summarizing the estimates from fitting our model to the time series for each of the four drug types over 23 years for each of the 48 contiguous states and the District of Columbia (DC). In Fig 1, we show posterior median estimates of death rates involving each substance by state with time series plots arranged approximately geographically. In Fig 2, we show posterior median estimates of the death rate for each state by involvement of each substance. The plot is ordered by CMS region so it roughly goes from states in the Northeast at the top to the West at the bottom. These estimates are also shown in traditional time series line plots in S1 Fig and by region in S2–S11 Figs. In general, the overall patterns approximately follow the three waves with death rates involving prescription opioids greatest before 2010, heroin greatest from 2010 to 2015, and synthetic opioids increasing since 2015. One of the most striking takeaways is the relative magnitude of death rates involving synthetic opioids since 2015 compared to the other drug types. It is clear how much more severe the overdose epidemic has become since 2015 with synthetic opioids relative to the initial wave driven by prescription opioid overdoses. While these general trends tend to hold across most states, we observe variability in the timing and severity across the country.

In northeast and mid-Atlantic states (regions 1, 2, and 3), we observe the most pronounced three wave structure of the opioid overdose epidemic. Rates of prescription-involved deaths are fairly similar across the 23 years with the exception of West Virginia where rates are notably higher than the rest of the region and the country (Figs 1 and 2). The second wave in this region can most clearly be observed in Fig 2 with higher rates of heroin-involved deaths starting just before 2015. Rates of heroin-involved deaths were highest between 2015 and 2020 in Massachusetts, Connecticut, New Jersey, Delaware, Maryland, DC, and West Virginia. Starting in 2015, we observe a rapid increase in rates of deaths involving synthetic opioids across all states in the region. Most striking are rates in DC and West Virginia that are approximately 20 deaths per 100,000 residents greater than other states in the region in 2021. Relative to the rest of the country, this region is, collectively, the most affected by the heroin and synthetic opioid waves of the epidemic. In most states in this region, rates of deaths involving unspecified drugs decreased over time. However, Pennsylvania had notably higher rates of unspecified drugs than other states, which may reflect differences in reporting [22, 23], a point we return to in the discussion below.

In southern and central states (regions, 4, 5, 6, and 7), we observe similar patterns to the northeast and mid-Atlantic, but generally see lower death rates and later onset of the second and third waves (Figs 1 and 2). Particularly in southern states, the heroin wave of the epidemic is less pronounced than in the northeastern states described earlier. Exceptions to these general patterns are Kentucky, Tennessee, and Ohio which appear more similar to the patterns observed in West Virginia, albeit less severe. This is not surprising as these states are all part of Appalachia and are demographically similar. Additionally, Texas, Kansas, Nebraska, and Arkansas have relatively low reported death rates across drug types and do not appear to experience increases associated with the three waves. New Mexico and Oklahoma both stand out for having relatively high rates of prescription-involved deaths, but while New Mexico experienced increases in heroin-involved deaths after 2010, both states have not yet experienced the third wave of synthetic opioid-involved deaths on the scale of some of the eastern states. In this region, we also see that New Mexico has relatively high rates of deaths involving unspecified drugs before 2010, and Louisiana has relatively high rates throughout the time period. In Louisiana in particular, it appears there may be substantial differences in reporting as death rates involving unspecified drugs are higher than the other three categories until the very end of the study period when it is surpassed by rates involving synthetic opioids.

In western states (regions 8, 9, and 10), we generally observe lower death rates across all drug types and see less evidence for the three waves (Figs 1 and 2). Across most states in this region, the prescription-involved death rates are fairly constant over the study period with higher rates observed in Utah and Nevada. We see little evidence of a second wave of heroin-involved overdose in this region and the third wave involving synthetic opioids appears to be just beginning at the end of the study period in 2021. One exception is Arizona which has higher rates of synthetic opioid-involved deaths that are more similar to its neighbor New Mexico than to the rest of the western portion of the country. We also observe relatively low rates across drug types that do not substantially increase over time in North Dakota, South Dakota, Montana, and Idaho. In general, the patterns observed in the western states are quite different than those in the eastern states. This is made particularly clear in Fig 2 as the intensity of the colors decreases moving down the rows of the figure from the eastern to western states.

We also show the posterior median estimated death rates and the relative risk comparing the death rate of the current year to the year prior as an animated time series of maps in S1 and S2 Videos. In each map, the outline indicates at least a 90% posterior probability that the rate increased (red) or decreased (blue) from the previous year. The maps make clear the regional patterns suggested by Figs 1 and 2. In S12 Fig, we show the posterior median estimates of the log odds of a regime change at each year for every state. Points colored red and blue to reflect at least a 90% probability of an increase and decrease in the death rate, respectively. Greater log odds indicate a higher probability of a regime change (i.e., more volatility in the death rate). These plots again reinforce the temporal ordering of the waves of the epidemic and illustrate the volatility and increasing rates of deaths involving synthetic opioids in the most recent years. We also notice the greatest log odds of a change point for increases in deaths involving synthetic opioids is in 2020, which coincided with the onset of the COVID-19 pandemic.

## Discussion

Our study highlights state-level variation in the timing and severity of the waves of the opioid epidemic by presenting a 23-year natural history of opioid overdose mortality in the US. Our statistical model offers a novel and flexible approach to inferring the location of multiple unknown changepoints and estimating a state-level time series of unknown functional form. Our findings reinforce general trends that are consistent with prior reports that document an ever-worsening overdose crisis, characterized by three waves [13, 24, 25] but illustrate the impacts of each wave varied by state. While the coarse timing of each wave is consistent with existing literature, we find state-level variation in the onset of each wave with prescription deaths beginning in 1999–2005, heroin deaths in 2010–2014, and synthetic opioid deaths in 2014–2021. The substantial variability around the timing of onset and the severity of each wave has not been previously reported. Through our model, we are able to assess and quantify this variability to improve our understanding of how and when rates of overdose deaths have changed across the country and what types of opioids have been involved. Our approach can be used to track a range of correlated indicators of drug-related harms to identify changes over time and describe spatial variability that can offer unique insights to researchers and health agencies as the overdose crisis continues to evolve.

The substantial elevation in overdose death rates over the three waves may reflect the unintended consequences of supply-side strategies implemented in the absence of concurrent demand-side strategies. In this case, there was substantial investment at the federal, state and local levels in policies and programs to regulate opioid prescribing and reduce harms related to the use of opioids in ways other than medically intended. These measures were enacted in a context of substantial policy and geographic constraints to the availability and access to opioid use disorder treatment and harm reduction services. While these policies did reduce high-risk forms of prescribing such as high-dose and long-duration prescriptions, and did stabilize overdoses involving prescription opioids, they likely also had unintended consequences in contributing to the shift towards the illegal market and the creation of ever-cheaper and more potent synthetic opioid products.

Previously reported patterns of the evolution of the opioid overdose epidemic obscured important heterogeneity. In our approach, we find West Virginia was a clear outlier in terms of prescription opioid overdose deaths, with prescription opioid overdose death rates that were more than double those of any other state, and this rate remained high until experiencing slight reductions in 2015. At its peak, DC experienced the highest rise in heroin-involved overdoses, and West Virginia, DC, and Ohio exhibited the highest rates of overdose involving synthetic opioids. The pattern of geographic spread of overdoses also differed across waves. Most states across the country experienced increases in prescription opioid overdose deaths from the beginning of the study period, but rates were particularly high in Central Appalachia. In contrast, increases in heroin and synthetic opioid overdoses were particularly notable on the East Coast, and then spread, to a lesser degree, throughout the country a couple of years later. These distinct patterns of geographic spread may reflect differences in the geography of the supply and social drivers of each wave. Aggressive marketing across the country of opioids as a safe, non-addictive alternative to treat noncancer chronic pain contributed to the rise in prescription opioid overdoses across the country, while the concentration of deindustrialization, increasingly precarious working conditions, and workplace injuries may have led to a particularly high rise in prescription opioid overdose deaths in places like Central Appalachia [26–29]. As state restrictions on the prescription opioid supply, and subsequent declines in opioid prescribing led to declines in prescription opioid use [30], a rise in the supply and decline in price of Colombian heroin [8], and a subsequent introduction of fentanyl [7] (often mixed with heroin or sold as counterfeit prescription opioids) into the East Coast drug market may have contributed to the initial rise in heroin and synthetic opioid overdoses in the Eastern region of the United States.

Our study also highlights that not all regions of the country followed the typically described “three waves” of the overdose crisis. For example, certain regions of the country did not see large changes in prescription opioid overdose deaths, but did see a later rise in overdoses involving heroin and synthetic opioids (i.e., New York, New Jersey, Missouri, and the Upper Midwest). Other regions like the Northwest exhibited increases in prescription opioid overdose deaths, but experienced consistently low rates of heroin and synthetic opioid overdose deaths throughout the study period. These patterns raise important questions about the factors that may have protected certain regions from experiencing the effects of certain waves of the opioid crisis or signs of waves that are still to come in the future, like the fourth wave involving stimulants and synthetic opioids [31–34]. Identifying such drivers may lead to critical insights about targets for prevention and policy investment. However, while variation can lead to actionable insights, it also the difficulty of controlling risks and studying what interventions may be effective because each state is facing their own unique version of the larger crisis.

At a larger level, the study findings showing heterogeneity in the timing and experience of the three waves across states and regions highlight the need for a locally-tailored response to the overdose crisis. Modeling approaches such as the one proposed here can be used to aid local jurisdictions in understanding how their own overdose profile has evolved and how it relates to the overdose profile in surrounding jurisdictions, so that local partners can identify the optimal interventions to address the local overdose problem.

The variation in findings by state and time supports the need for disaggregated, flexible modeling of overdose trends, which was made possible by our innovative approach. Prior work describing the geographic and temporal spread of overdose has been limited by small counts, and by a need to pre-specify the number and/or location of change points, or use approaches like joinpoint modeling [35] that rely on repeated statistical significance testing. Our models overcame these issues through use of a multivariate Bayesian hierarchical model, which allows information-sharing across regions and full propagation of uncertainty to inform inferences about the timing and magnitude of changes. Further, our approach captures the complex trends of overdose without making parametric assumptions about the shape of the time series or prespecifying the number of change points. This allowed us to estimate and illustrate changes in death rates by state, thus tracking the evolution of the overdose waves and highlighting periods of volatility and stability of the overdose epidemic. This enabled us to focus on structural shifts in the overdose rates and smooth over natural year-to-year variation, which can be challenging to determine from simply descriptively examining death rates. These methods can be used to track the evolution of other public health issues that are pervaded by complex temporal trajectories and exhibit localized patterns of variation.

Study findings should be considered in light of the following limitations. First, we capture trends in reported opioid overdose deaths—the considerable devastating consequences of the addiction and overdose crisis beyond opioid deaths are thus not considered. Second, heterogeneity at finer geographic levels is quite likely [36]. However, small sample constraints become even more acute at smaller levels of resolution. Third, substantial variation can exist across space and time in the reporting of the involvement of specific drugs in the death certificates [37]. However, this is much less of a concern at the state level. Further, we examined trends in overdoses involving unspecified drugs to address this problem, and highlighted states where patterns differed.

While we included overdoses involving unspecified drugs to attempt to gain some insights on reporting variation, further examination of potential differential reporting is warranted. There are many potential drivers of reporting heterogeneity that we were not able to address including autopsy coverage for overdoses, political incentives, and accessibility of public health funding that may be allocated based on overdose rates [38]. An improved understanding of sources of reporting variation across the country would enhance our ability to interpret trends in existing data on reported overdose deaths [22, 23].

## Conclusion

The United States is in the midst of an ever-worsening overdose epidemic. While the country overall has experienced three general waves of opioid overdose, each one at higher rates than the preceding wave, substantial heterogeneity exists across states. Through a single statistical model, we have facilitated comparison and exploration of this heterogeneity by compiling a comprehensive state-level natural history of the epidemic in the United States. This highlighted that certain states experienced rates of overdose that were double those of any other state, yet other states and regions avoided entire waves of the opioid overdose crisis. Such variation across space and time suggests that a universal federal solution may not exist and highlights the need for a localized response, that addresses the specific social environmental and policy drivers that contributed to the rise in overdose rates in a specific state and time. Concurrently, stable, low rates of overdose in certain states in the midst of a national epidemic raise important questions about the factors and policies that can protect communities from a rise in overdose. Such insights could prove instrumental in guiding future effective and localized policy responses to this national crisis. Describing and improving our understanding of this heterogeneity is an initial step to customizing policies and interventions to the specific environment experienced by states that shape their own unique version of the overdose crisis.

## Supporting information

S1 Table CMS regions. States within each Centers for Medicare and Medicaid Services (CMS) Region.
(PDF)

S1 Appendix Statistical details. Detailed specification of the statistical model.
(PDF)

S1 File R code. R code file to implement the statistical model used.
(PDF)

S1 Fig State-level time series. Posterior median estimates of the overdose death rates per 100,000 residents related to each drug type from 1999-2021 for each state. Each time series is colored by the Centers for Medicare and Medicaid Services (CMS) region of the state.
(TIF)

S2 Fig CMS Region 1: State-level time series. Posterior median estimates of the overdose death rates per 100,000 residents related to each drug type from 1999-2021 for each state in CMS Region 1. Each time series is colored by the state.
(TIF)

S3 Fig CMS Region 2: State-level time series. Posterior median estimates of the overdose death rates per 100,000 residents related to each drug type from 1999-2021 for each state in CMS Region 2. Each time series is colored by the state.
(TIF)

S4 Fig CMS Region 3: State-level time series. Posterior median estimates of the overdose death rates per 100,000 residents related to each drug type from 1999-2021 for each state in CMS Region 3. Each time series is colored by the state.
(TIF)

S5 Fig CMS Region 4: State-level time series. Posterior median estimates of the overdose death rates per 100,000 residents related to each drug type from 1999-2021 for each state in CMS Region 4. Each time series is colored by the state.
(TIF)

S6 Fig CMS Region 5: State-level time series. Posterior median estimates of the overdose death rates per 100,000 residents related to each drug type from 1999-2021 for each state in CMS Region 5. Each time series is colored by the state.
(TIF)

S7 Fig CMS Region 6: State-level time series. Posterior median estimates of the overdose death rates per 100,000 residents related to each drug type from 1999-2021 for each state in CMS Region 6. Each time series is colored by the state.
(TIF)

S8 Fig CMS Region 7: State-level time series. Posterior median estimates of the overdose death rates per 100,000 residents related to each drug type from 1999-2021 for each state in CMS Region 7. Each time series is colored by the state.
(TIF)

S9 Fig CMS Region 8: State-level time series. Posterior median estimates of the overdose death rates per 100,000 residents related to each drug type from 1999-2021 for each state in CMS Region 8. Each time series is colored by the state.
(TIF)

S10 Fig CMS Region 9: State-level time series. Posterior median estimates of the overdose death rates per 100,000 residents related to each drug type from 1999-2021 for each state in CMS Region 9. Each time series is colored by the state.
(TIF)

S11 Fig CMS Region 10: State-level time series. Posterior median estimates of the overdose death rates per 100,000 residents related to each drug type from 1999-2021 for each state in CMS Region 10. Each time series is colored by the state.
(TIF)

S12 Fig Log odds of a changepoint. Posterior median estimates of the region-specific average log odds of a change in the mean overdose death rate for each state and drug type from 2000-2021.
(TIF)

S1 Video Animated death rate maps. Maps showing the posterior median overdose death rate per 100,000 residents for deaths involving each drug type from 1999-2021. Red outlines reflect a posterior probability of greater than 0.9 that the rate increased from the previous year, and blue outlines reflect a posterior probability of greater than 0.9 that the rates decreased from the previous year.
(GIF)

S2 Video Animated relative risk maps. Maps showing the posterior median log relative risk of death for the current year compared to the previous year for deaths involving each drug type from 2000-2021. Red outlines reflect a posterior probability of greater than 0.9 that the rate increased from the previous year, and blue outlines reflect a posterior probability of greater than 0.9 that the rates decreased from the previous year.
(GIF)
