# Supplementary material for: A state-level history of opioid overdose deaths in the United States: 1999-2021
Source: PLoS One. 2024 Sep 6;19(9):e0309938. doi: 10.1371/journal.pone.0309938 (PMC11379184; doi:10.1371/journal.pone.0309938)
Supplement: S1 Table — States within each Centers for Medicare and Medicaid Services (CMS) Region. (PDF) [file pone.0309938.s001.pdf]

S1 Table: A state-level history of opioid overdose deaths in the United States: 1999-2021

David Kline*[1], Staci A. Hepler[2], Noa Krawczyk[3], Ariadne Rivera-Aguirre[3], Lance A. Waller[4], Magdalena Cerdá[3],

1 Department of Biostatistics and Data Science, Division of Public Health Sciences, Wake Forest University School of Medicine, Winston-Salem, North Carolina, United States
2 Department of Statistical Sciences, College of Arts and Sciences, Wake Forest University, Winston-Salem, North Carolina, United States
3 Center for Opioid Epidemiology and Policy, Division of Epidemiology, Department of Population Health, New York University Grossman School of Medicine, New York, New York, United States
4 Department of Biostatistics and Bioinformatics, Rollins School of Public Health, Emory University, Atlanta, Georgia, United States

* dkline@wakehealth.edu

S1 Table

Region	States
1	Connecticut, Maine, Massachusetts, New Hampshire, Rhode Island, Vermont
2	New Jersey, New York
3	Delaware, District of Columbia, Maryland, Pennsylvania, Virginia, West Virginia
4	Alabama, Florida, Georgia, Kentucky, Mississippi, North Carolina, South Carolina, Tennessee
5	Illinois, Indiana, Michigan, Minnesota, Ohio, Wisconsin
6	Arkansas, Louisiana, New Mexico, Oklahoma, Texas
7	Iowa, Kansas, Missouri, Nebraska
8	Colorado, Montana, North Dakota, South Dakota, Utah, Wyoming
9	Arizona, California, Nevada
10	Idaho, Oregon, Washington

Table 1. States within each Centers for Medicare and Medicaid Services (CMS) Region.
