# Supplementary material for: A state-level history of opioid overdose deaths in the United States: 1999-2021
Source: PLoS One. 2024 Sep 6;19(9):e0309938. doi: 10.1371/journal.pone.0309938 (PMC11379184; doi:10.1371/journal.pone.0309938)
Supplement: S1 Appendix — Detailed specification of the statistical model. (PDF) [file pone.0309938.s002.pdf]

S1 Appendix: A state-level history of opioid overdose deaths in the United States: 1999-2021

David Kline*[1], Staci A. Hepler[2], Noa Krawczyk[3], Ariadne Rivera-Aguirre[3], Lance A. Waller[4], Magdalena Cerdá[3],

1 Department of Biostatistics and Data Science, Division of Public Health Sciences, Wake Forest University School of Medicine, Winston-Salem, North Carolina, United States
2 Department of Statistical Sciences, College of Arts and Sciences, Wake Forest University, Winston-Salem, North Carolina, United States
3 Center for Opioid Epidemiology and Policy, Division of Epidemiology, Department of Population Health, New York University Grossman School of Medicine, New York, New York, United States
4 Department of Biostatistics and Bioinformatics, Rollins School of Public Health, Emory University, Atlanta, Georgia, United States

* dkline@wakehealth.edu

Statistical Model

The main objective of the analysis is to describe changes in overdose death rates involving prescription opioids, heroin, synthetic opioids, and unspecified narcotics across space and time. To do so, we develop a model that allows us to estimate state-specific time series for death rates involving each drug type and allows for an unknown number of change points. Our model provides a flexible framework for estimating each time series, allows for full quantification of uncertainty within a unified model through Bayesian inference, and allows us to account for multivariate dependence across drug types.

Data Model

Let $Y_{st}^{(d)}$ be the observed count of overdose deaths involving drug $d = 1, ..., D$ in state s during year t. We assume

$$Y_{st}^{(d)} | \theta(s)^{(d)}_{r_t(s,d)} \overset{ind}{\sim} \text{Poisson}\left(P_{st} \cdot \theta(s)^{(d)}_{r_t(s,d)}\right)$$

where P_{st} in the population of state s in year t. We define $r_t(s,d) \in \{1, 2, ..., T\}$ as the regime at time t for state s and drug type d. Each regime is specific to a state and drug type and is not meaningfully comparable across states or drug types. Note that $r_{t+1}(s,d)$ is either equal to $r_t(s,d)$ or $r_t(s,d) + 1$ and a change in regime from time t to $t+1$ in state s for drug type d means that the death rate is different in year $t+1$ from year t within that state and drug type. For every state, drug, and time, the regime label $r_t(s,d)$ defines the regime-state-drug-specific death rate, $\theta(s)^{(d)}_{r_t(s,d)}$. Note that $\theta(s)^{(d)}_{r_t(s,d)}$ is constant for all years t that are in the same regime.

Process Model

The process model consists of a model for the drug-specific death rates for each regime within each state and a model for transitioning to a new regime. For the death rates, let

$$\theta(s)^{(d)}_{r_t(s,d)} \overset{ind}{\sim} \text{Gamma}(\alpha_{sd}, \beta_{sd})$$

where α_{sd} and β_{sd} are state- and drug-specific hyperparameters. We assume there exists a latent binary process, $j^{(d)}_{ts}$, that indicates whether or not a jump to a new regime occurred in year t at state s for drug d. That is, for $t > 1$, let

$$r_t(s,d) = \begin{cases} r_{t-1}(s,d) & j^{(d)}_{ts} = 0 \\ r_{t-1}(s,d) + 1 & j^{(d)}_{ts} = 1, \end{cases}$$

which implies that the regime changes for drug d in state s at time t when $j^{(d)}_{ts} = 1$. We assume

$$j^{(d)}_{ts} | p^{(d)}_{ts} \overset{ind}{\sim} \text{Bernoulli}(p^{(d)}_{ts}),$$

and use a centered autologistic model for the jump probability such that

$$\text{logit}(p^{(d)}_{ts}) = \mu^{(d)}_{t,C(s)} + \rho^{(d)} \left(j^{(d)}_{ts} - \frac{\exp(\mu^{(d)}_{t,C(s)})}{1 + \exp(\mu^{(d)}_{t,C(s)})} \right), \quad (1)$$

where $\mu^{(d)}_{t,C(s)}$ is a regional random effect [1]. For each year t and region $C(s)$, let $\boldsymbol{\mu}_{t,C(s)} = \left(\mu^{(1)}_{t,C(s)}, ..., \mu^{(D)}_{t,C(s)}\right)$. We assume

$$\boldsymbol{\mu}_{t,C(s)} \sim N_D(\boldsymbol{\mu}_t, \boldsymbol{\Sigma}),$$

where $\boldsymbol{\mu}_t = (\mu^{(1)}_t, ..., \mu^{(D)}_t)$ and $\mu^{(d)}_t$ reflects the national average log odds of a regime change for drug d at time t and $\boldsymbol{\Sigma}$ is a $D \times D$ dimensional covariance matrix to quantify dependence between drug types. Intuitively, we can think of $p^{(d)}_{ts}$ as a measure of volatility in the death rates. That is, the higher the probability of a regime change the more volatile, or less stable, the death rate is from one year to the next. The regional random effect implies that all states within the same region share an intercept, and the model for that random effect accounts for dependence among drug types. The centered autologistic model accounts for the possibility that the probability of a regime change in a state may be impacted by past volatility in that state.

Prior Model and Computation

Since we are fitting the model within the Bayesian paradigm, we must assign prior distributions to all parameters. We use proper but diffuse prior distributions for all parameters. More specifically, we assume $\alpha_{sd} \overset{iid}{\sim} \text{Exponential}(1)$ and $\beta_{sd} \overset{iid}{\sim} \text{Gamma}(0.1, 1)$. We assume $\mu^{(d)}_t \overset{iid}{\sim} N(0, 9)$. We also assume $\rho^{(d)} \overset{iid}{\sim} \text{Uniform}(0, 1)$. Finally, we assume $\boldsymbol{\Sigma} \sim \text{Inverse Wishart}(\mathbf{I}_D, D + 1)$ where $\mathbf{I}_D$ is a $D \times D$ identity matrix. To fit the model, we ran a Markov chain Monte Carlo algorithm using `nimble` [2] in `R` for 500,000 iterations. We discarded the first 250,000 as burn-in and thinned the remaining samples by 50. Convergence was assessed visually using trace plots. Computation took approximately 5.5 hours using the DEAC cluster [3].

References

1. Caragea PC, Kaiser MS. Autologistic models with interpretable parameters. Journal of Agricultural, Biological, and Environmental Statistics. 2009;14(3):281–300.

2. de Valpine P, Turek D, Paciorek CJ, Anderson-Bergman C, Temple Lang D, Bodik R. Programming with Models: Writing Statistical Algorithms for General Model Structures with NIMBLE. Journal of Computational and Graphical Statistics. 2017;26:403–417. doi:10.1080/10618600.2016.1172487.

3. Information Systems and Wake Forest University. WFU High Performance Computing Facility; 2021. Available from: https://hpc.wfu.edu.
