# Supplementary material for: A state-level history of opioid overdose deaths in the United States: 1999-2021
Source: PLoS One. 2024 Sep 6;19(9):e0309938. doi: 10.1371/journal.pone.0309938 (PMC11379184; doi:10.1371/journal.pone.0309938)
Supplement: S1 File — R code file to implement the statistical model used. (PDF) [file pone.0309938.s003.pdf]

```
###################################################################
#####
#####
###################################################################

library(nimble)
library(tidyverse)
library(dplyr)
library(spdep)
library(maps)
library(maptools)
library(classInt)
library(data.table)


###### load the data

load('Data2Run.Rda')

###### this loads a data frame named data with columns for
###### State, Year, Population, deaths for each drug type of interest
###### CMS region

y =
cbind(data$RxOpioidDeathNoSynthCount,data$HeroinDeathCount,data$SyntheticDeathCount,data$UnspecifiedDeathCount)
off = data$Population

S = length(unique(data$State))
T = length(unique(data$Year))
D = dim(y)[2]

data$State = toupper(data$State)

##Create an adjacency matrix for the states in the US

usa.state = maps::map(database="state", fill=TRUE, plot=FALSE)
state.ID <- sapply(strsplit(usa.state$names, ":"), function(x) x[1])

st_crosswalk <- tibble(state = state.name) %>%
  bind_cols(tibble(abb = state.abb)) %>%
  bind_rows(tibble(state = "District of Columbia", abb = "DC"))

names(st_crosswalk)[2]="State"
stateinfo = left_join(data[1:S,c(1,8)],st_crosswalk,by="State")


### get the map then remove Hawaii and Alaska and re-order according to abbreviation

usa.poly = map2SpatialPolygons(usa.state, IDs=state.ID)
usa.nb = poly2nb(usa.poly)
usa.adj.mat = nb2mat(usa.nb, style="B")

usa.adj.mat =
usa.adj.mat[order(match(unique(state.ID),stateinfo$state)),order(match(unique(state.ID),stateinfo$state))]

##Write the 0-1 adjacency matrix
W = usa.adj.mat
W[(W>0)] = 1

num<-colSums(W)

adj<-NULL
for(j in 1:S){
  adj<-c(adj,which(W[j,]==1))
}
adj<-as.vector(adj)
num<-as.vector(num)
weights<-1+0*num

model_code=nimbleCode({
  for(s in 1:S){
    for(t in 1:T){
      for(d in 1:D){
        y[((t-1)*S+s),d] ~ dpois(P[((t-1)*S+s)]*theta[((t-1)*S+s),d])
        beta[((t-1)*S+s),d] ~ dgamma(aa[s,d],bb[s,d])
      }
    }
  }
```

```
  for(s in 1:S){
    for(d in 1:D){
      R[1,s,d] <- 1
      J[1,s,d] <- 1
      theta[s,d] <- beta[s,d]
      mu.p.s[1,s,d] <- 10
      for(t in 2:T){
        J[t,s,d] ~ dbern(p[t,s,d])
        mu.p.s[t,s,d] <- mu.p.reg[t,reg[s],d]
        logit(p[t,s,d]) <- mu.p.s[t,s,d] + rho.mu.p[d]*(J[t-1,s,d]-ilogit(mu.p.s[t-1,s,d]))
        R[t,s,d] <- R[t-1,s,d]+J[t,s,d]
        theta[((t-1)*S+s),d] <- theta[((t-2)*S+s),d]*(1-J[t,s,d]) + beta[((t-1)*S+s),d]*J[t,s,d]
      }
    }
  }

  ### prior for regional RE mu.p.reg
  for(t in 1:T){
    for(r in 1:num.reg){
      mu.p.reg[t,r,1:D] ~ dmnorm(mu.t[t,1:D],cov=Sig.p[1:D,1:D])
    }
  }
  for(t in 1:T){
    for(d in 1:D){
      mu.t[t,d] ~ dnorm(0,sd=3)
    }
  }
  Sig.p[1:D,1:D] ~ dwish(WisPsi[1:D,1:D],D+1)

  for(s in 1:S){
    for(d in 1:D){
      aa[s,d] ~ dexp(1.0)
      bb[s,d] ~ dgamma(0.1,1.0)
    }
  }
  for(d in 1:D){
    rho.mu.p[d] ~ dunif(0,1)
  }


})

mod_constants=list(T=T,P=off,S=S,D=D,num.reg = length(unique(data$CMS)),reg = data$CMS,adj=adj,num=num,WisPsi
= diag(D))

mod_data=list(y=y)

# Set initial values.
jump.init = c(1,rep(1,T-1))
jump.init = array(rep(jump.init,S*D),c(T,S,D))
Prec0.init = array(0,c(D,D,T))
for(t in 1:T){
  Prec0.init[,,t]=diag(D)
}
mod_inits=list(J = jump.init,beta = matrix(.0001,S*T,D),p = array(.5,c(T,S,D)),mu.p.s =
array(0,c(T,S,D)),mu.p.reg = array(0,c(T,length(unique(data$CMS)),D)),mu.t = matrix(0,T,D),Sig.p=diag(D))
nimble_model <- nimbleModel(model_code, mod_constants,mod_data,mod_inits)
compiled_model <- compileNimble(nimble_model,resetFunctions = TRUE)

# Set up samplers.
mcmc_conf <- configureMCMC(nimble_model,useConjugacy = TRUE)
mcmc_conf$addMonitors('theta','R','mu.p.s','mu.p.reg','Sig.p','p')

nimble_mcmc<-buildMCMC(mcmc_conf)
compiled_mcmc<-compileNimble(nimble_mcmc, project = nimble_model,resetFunctions = TRUE)

# Run the model
MCS=500000
s.t = Sys.time()
samples=runMCMC(compiled_mcmc,inits=mod_inits,
                nchains = 1, nburnin=MCS/2,niter = MCS,samplesAsCodaMCMC = TRUE,thin=50,
                summary = FALSE, WAIC = FALSE,progressBar=TRUE)
Sys.time()-s.t
save(samples,file="Output.Rda")

quit()
```
