# Supplementary figures and images for: A state-level history of opioid overdose deaths in the United States: 1999-2021
Source: PLoS One. 2024 Sep 6;19(9):e0309938. doi: 10.1371/journal.pone.0309938 (PMC11379184; doi:10.1371/journal.pone.0309938)

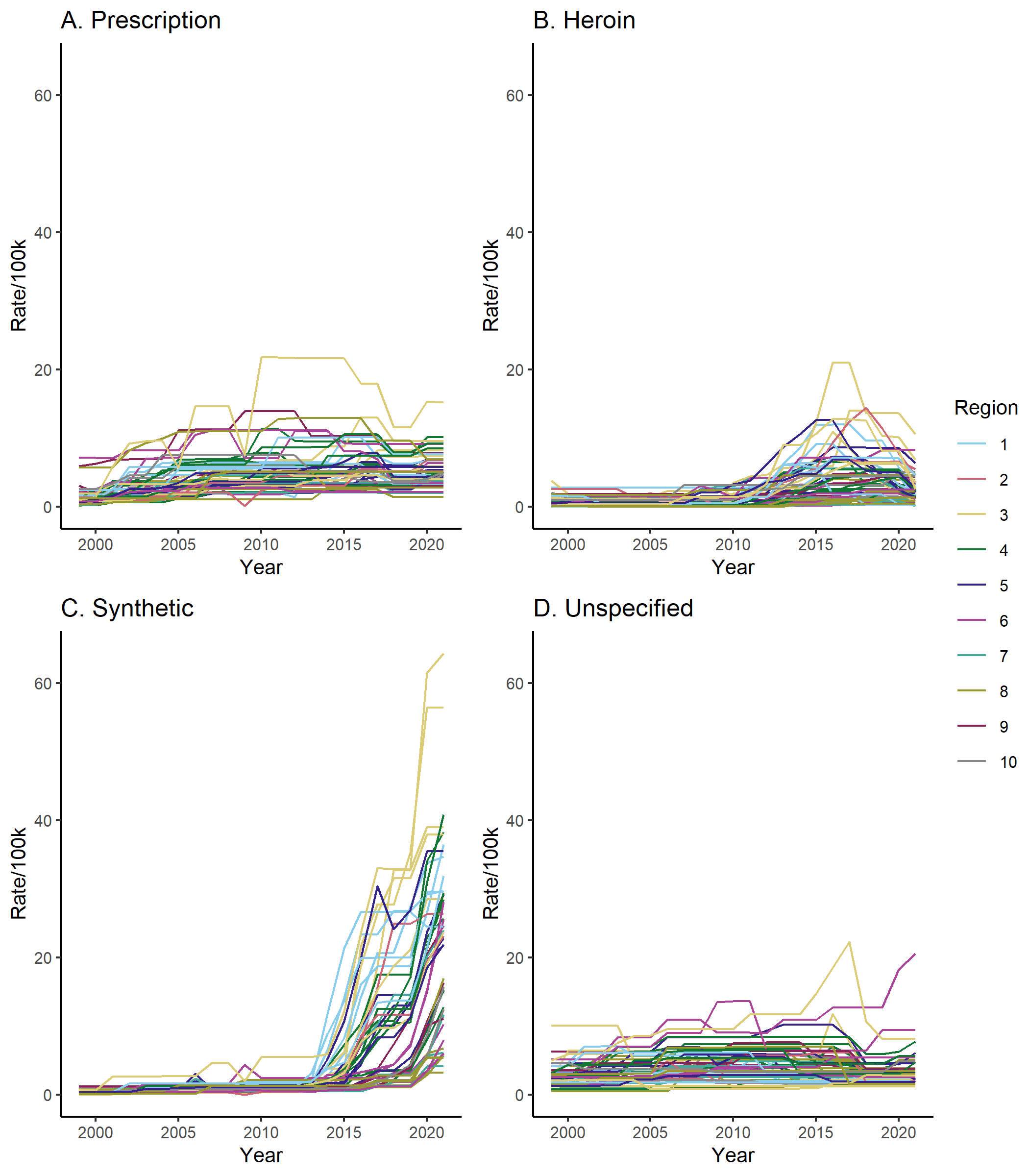

Supplement: S1 Fig — Posterior median estimates of the overdose death rates per 100,000 residents related to each drug type from 1999-2021 for each state. Each time series is colored by the Centers for Medicare and Medicaid Services (CMS) region of the state. (TIF) [file pone.0309938.s004.tif]

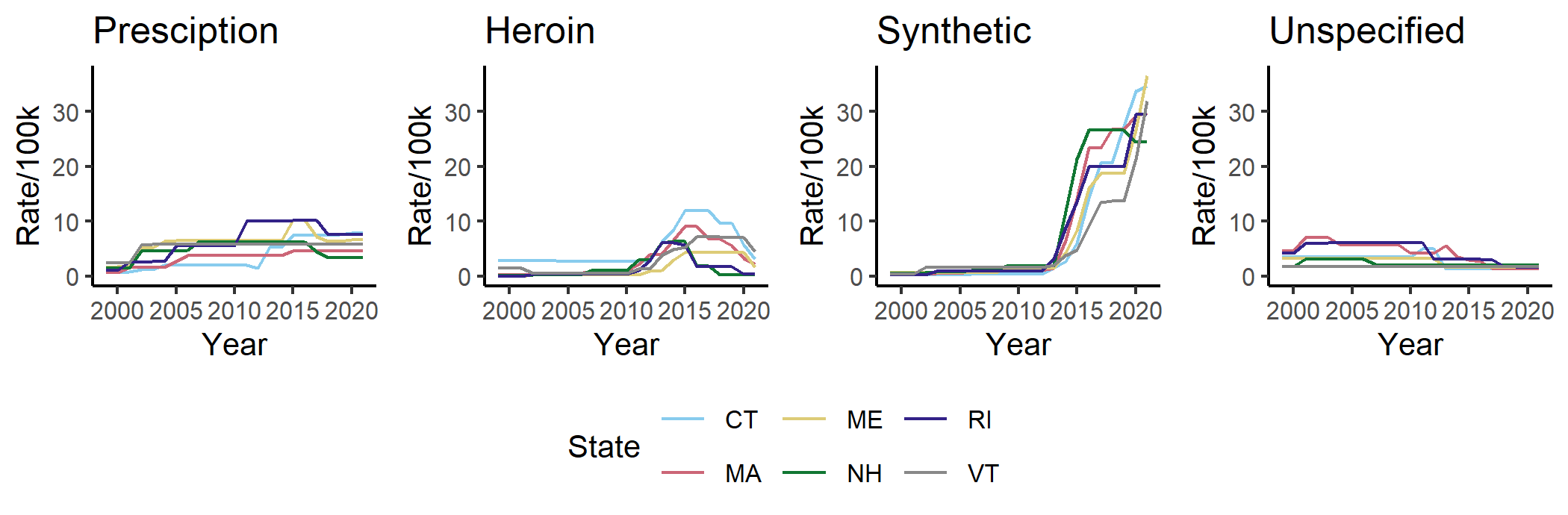

Supplement: S2 Fig — Posterior median estimates of the overdose death rates per 100,000 residents related to each drug type from 1999-2021 for each state in CMS Region 1. Each time series is colored by the state. (TIF) [file pone.0309938.s005.tif]

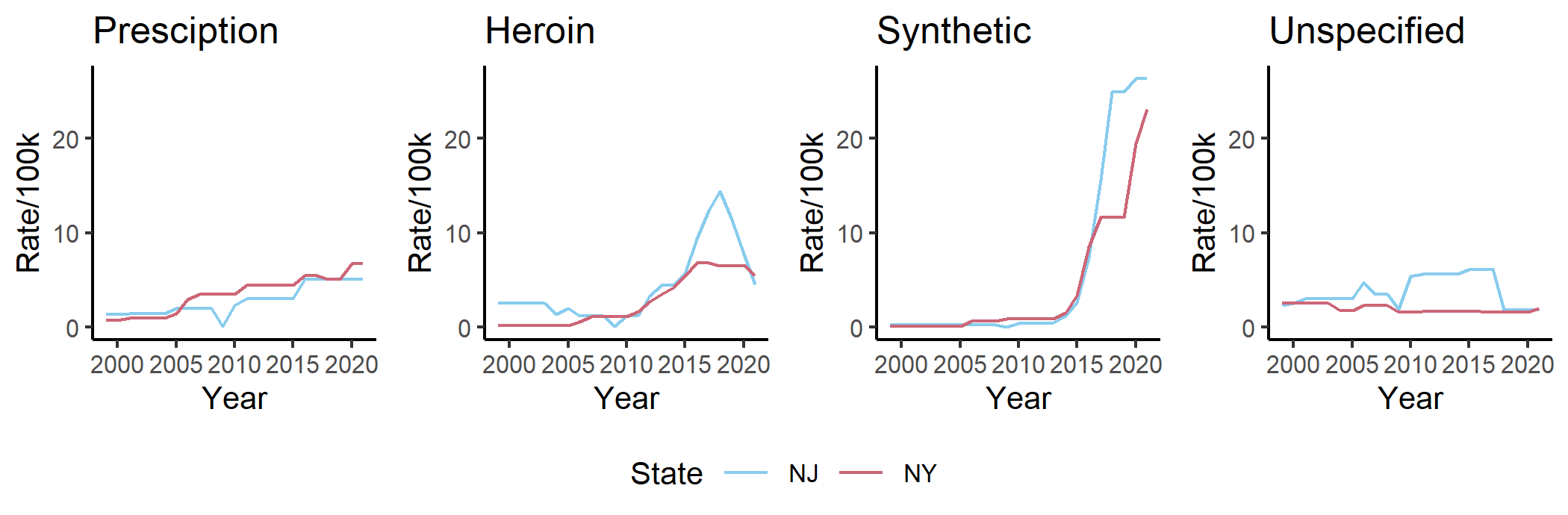

Supplement: S3 Fig — Posterior median estimates of the overdose death rates per 100,000 residents related to each drug type from 1999-2021 for each state in CMS Region 2. Each time series is colored by the state. (TIF) [file pone.0309938.s006.tif]

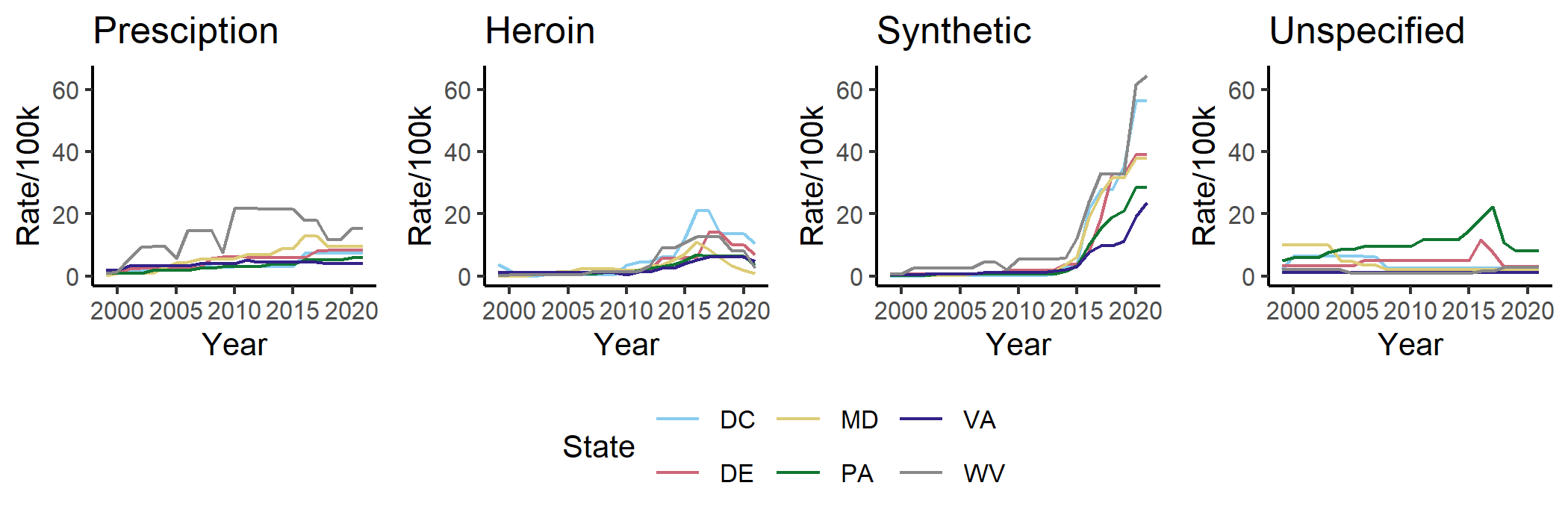

Supplement: S4 Fig — Posterior median estimates of the overdose death rates per 100,000 residents related to each drug type from 1999-2021 for each state in CMS Region 3. Each time series is colored by the state. (TIF) [file pone.0309938.s007.tif]

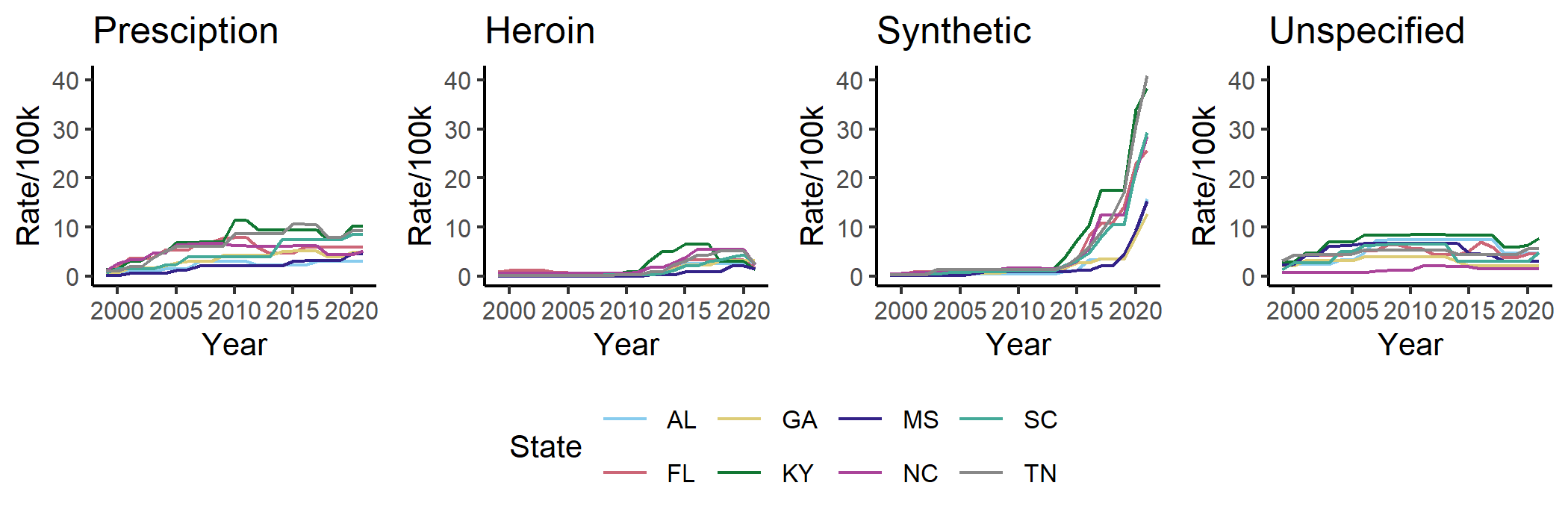

Supplement: S5 Fig — Posterior median estimates of the overdose death rates per 100,000 residents related to each drug type from 1999-2021 for each state in CMS Region 4. Each time series is colored by the state. (TIF) [file pone.0309938.s008.tif]

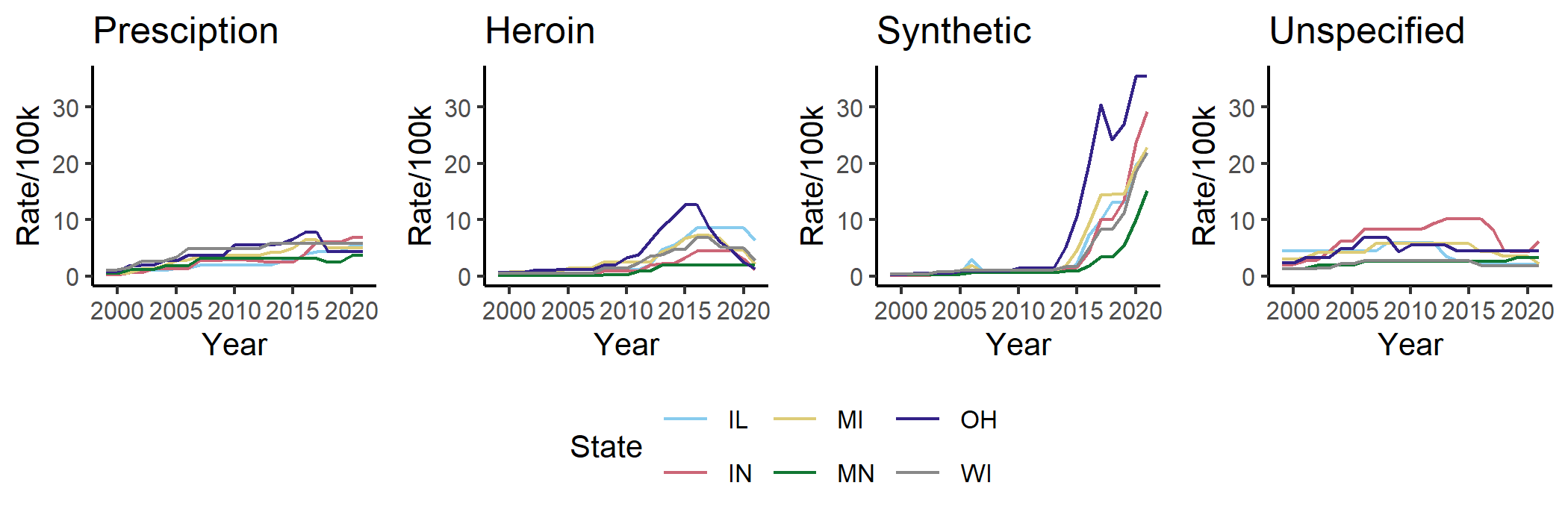

Supplement: S6 Fig — Posterior median estimates of the overdose death rates per 100,000 residents related to each drug type from 1999-2021 for each state in CMS Region 5. Each time series is colored by the state. (TIF) [file pone.0309938.s009.tif]

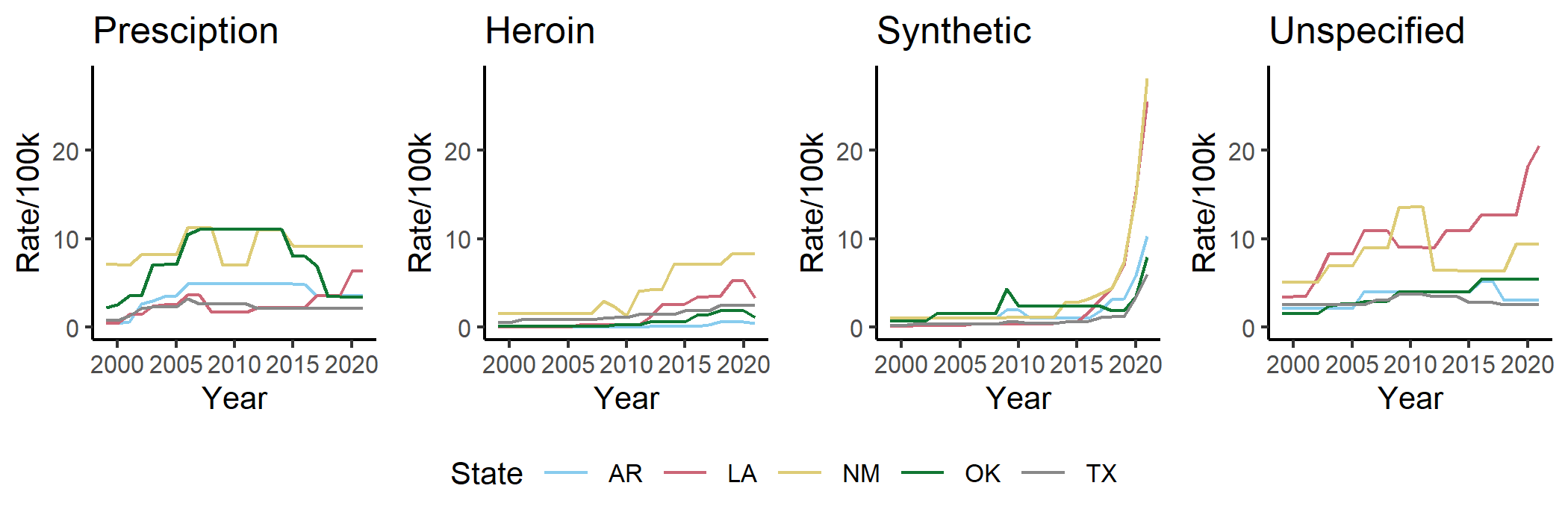

Supplement: S7 Fig — Posterior median estimates of the overdose death rates per 100,000 residents related to each drug type from 1999-2021 for each state in CMS Region 6. Each time series is colored by the state. (TIF) [file pone.0309938.s010.tif]

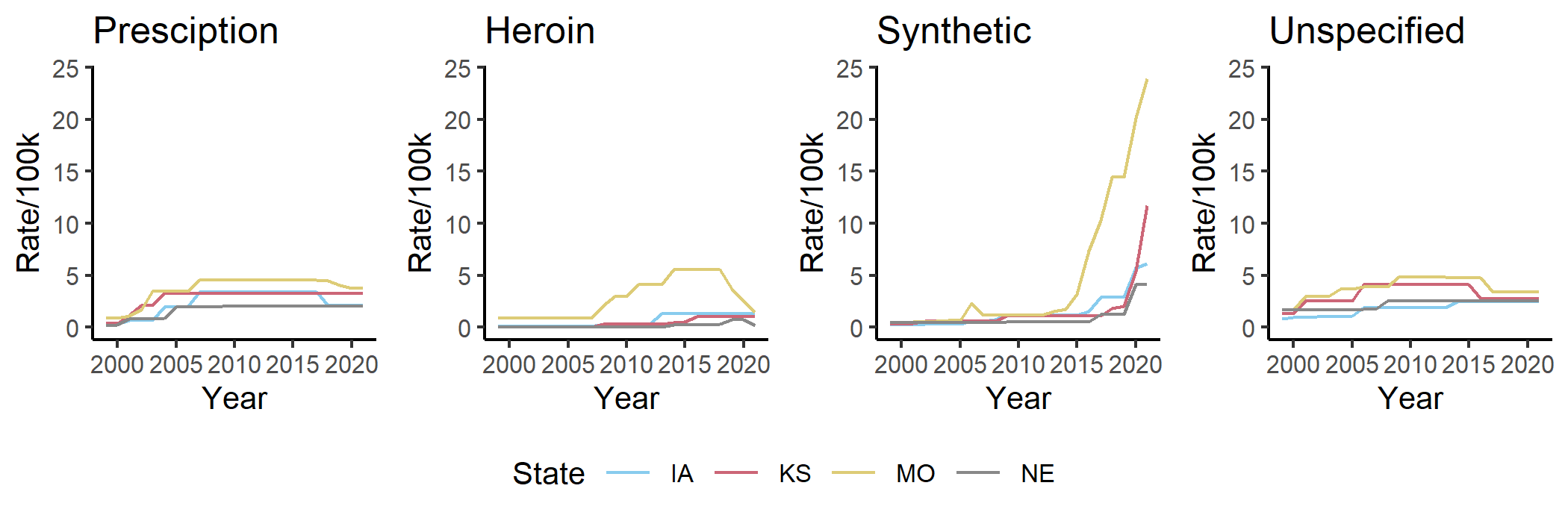

Supplement: S8 Fig — Posterior median estimates of the overdose death rates per 100,000 residents related to each drug type from 1999-2021 for each state in CMS Region 7. Each time series is colored by the state. (TIF) [file pone.0309938.s011.tif]

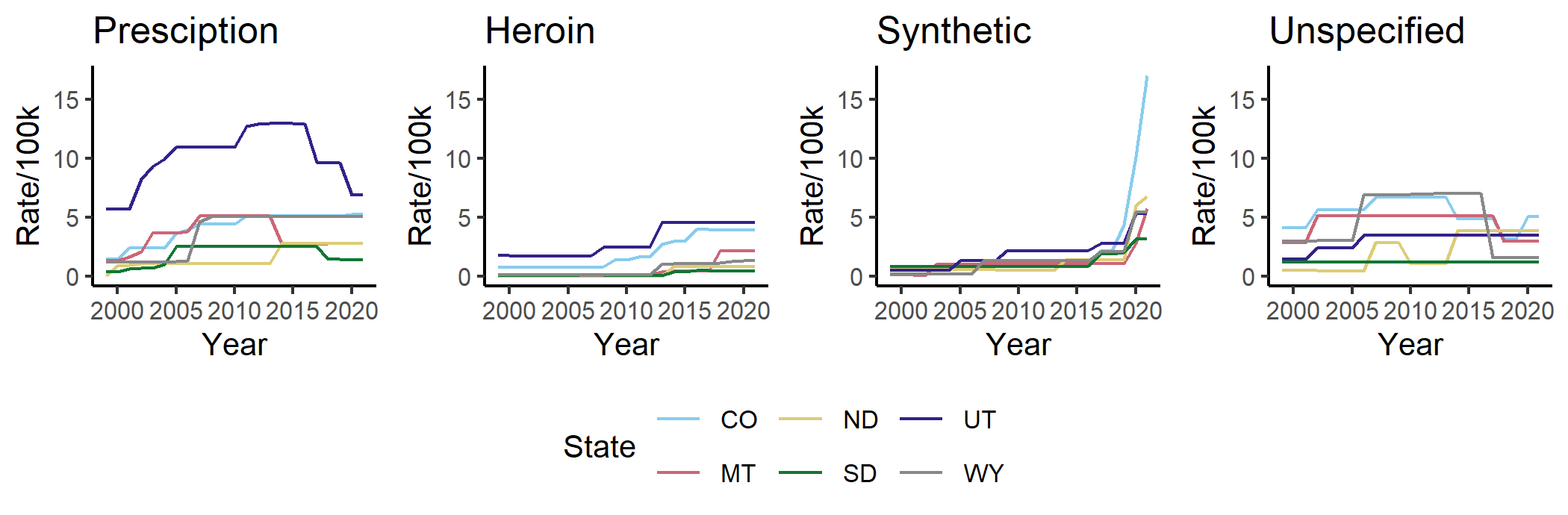

Supplement: S9 Fig — Posterior median estimates of the overdose death rates per 100,000 residents related to each drug type from 1999-2021 for each state in CMS Region 8. Each time series is colored by the state. (TIF) [file pone.0309938.s012.tif]

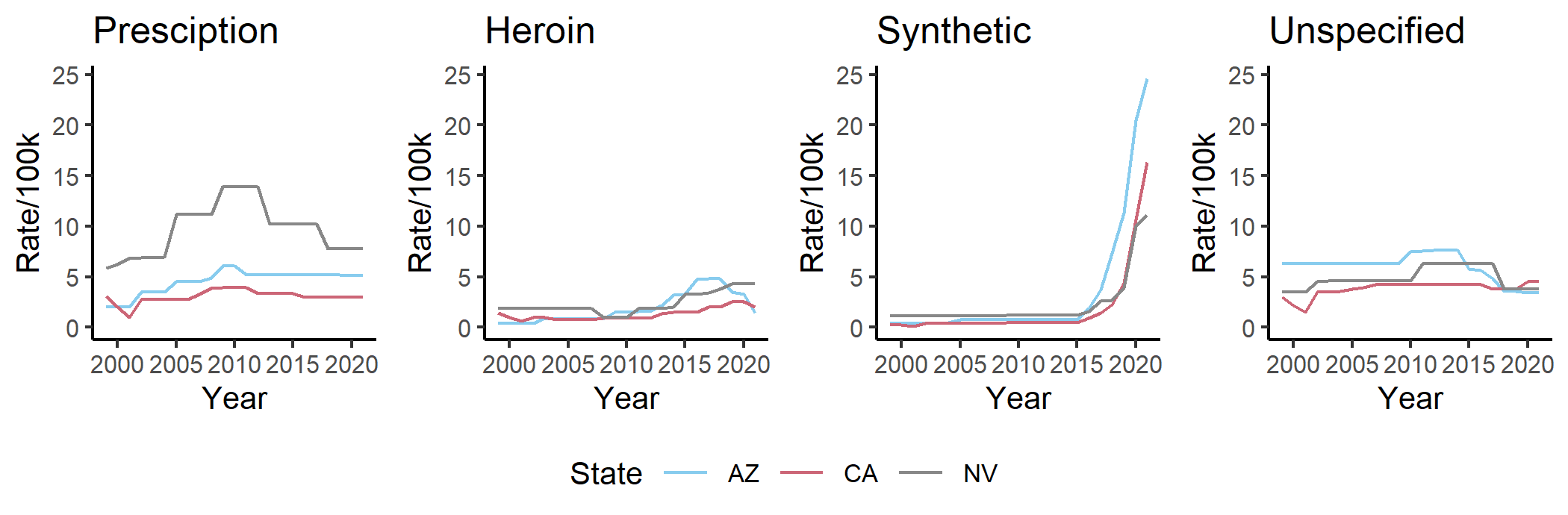

Supplement: S10 Fig — Posterior median estimates of the overdose death rates per 100,000 residents related to each drug type from 1999-2021 for each state in CMS Region 9. Each time series is colored by the state. (TIF) [file pone.0309938.s013.tif]

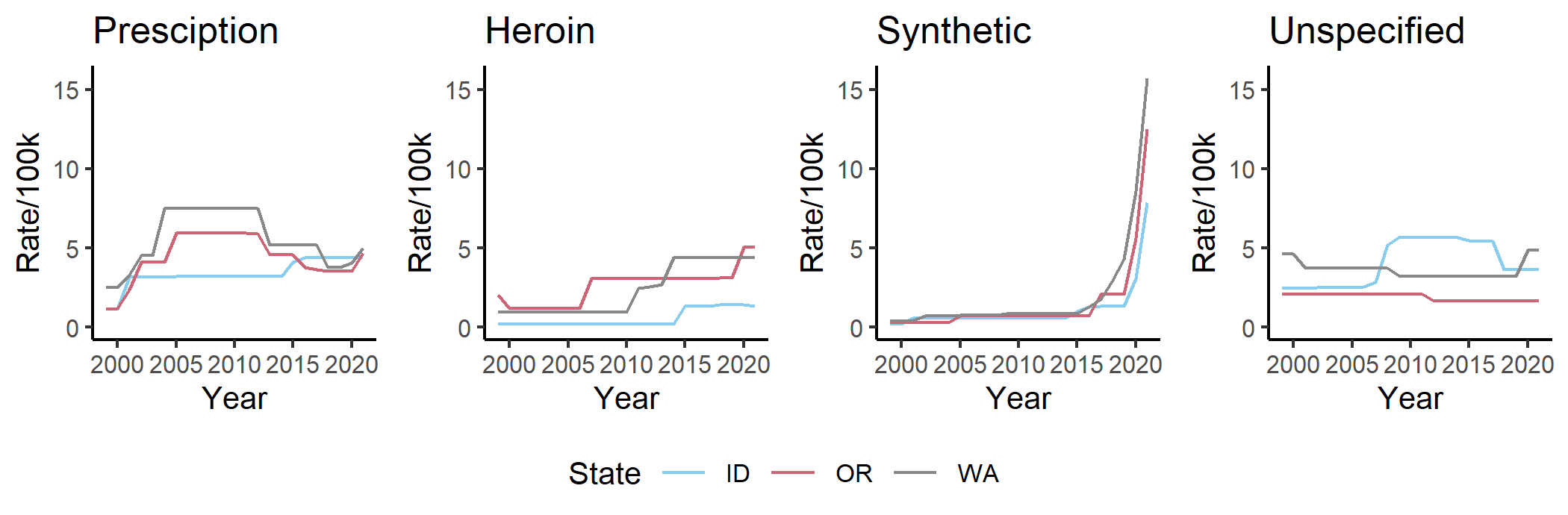

Supplement: S11 Fig — Posterior median estimates of the overdose death rates per 100,000 residents related to each drug type from 1999-2021 for each state in CMS Region 10. Each time series is colored by the state. (TIF) [file pone.0309938.s014.tif]

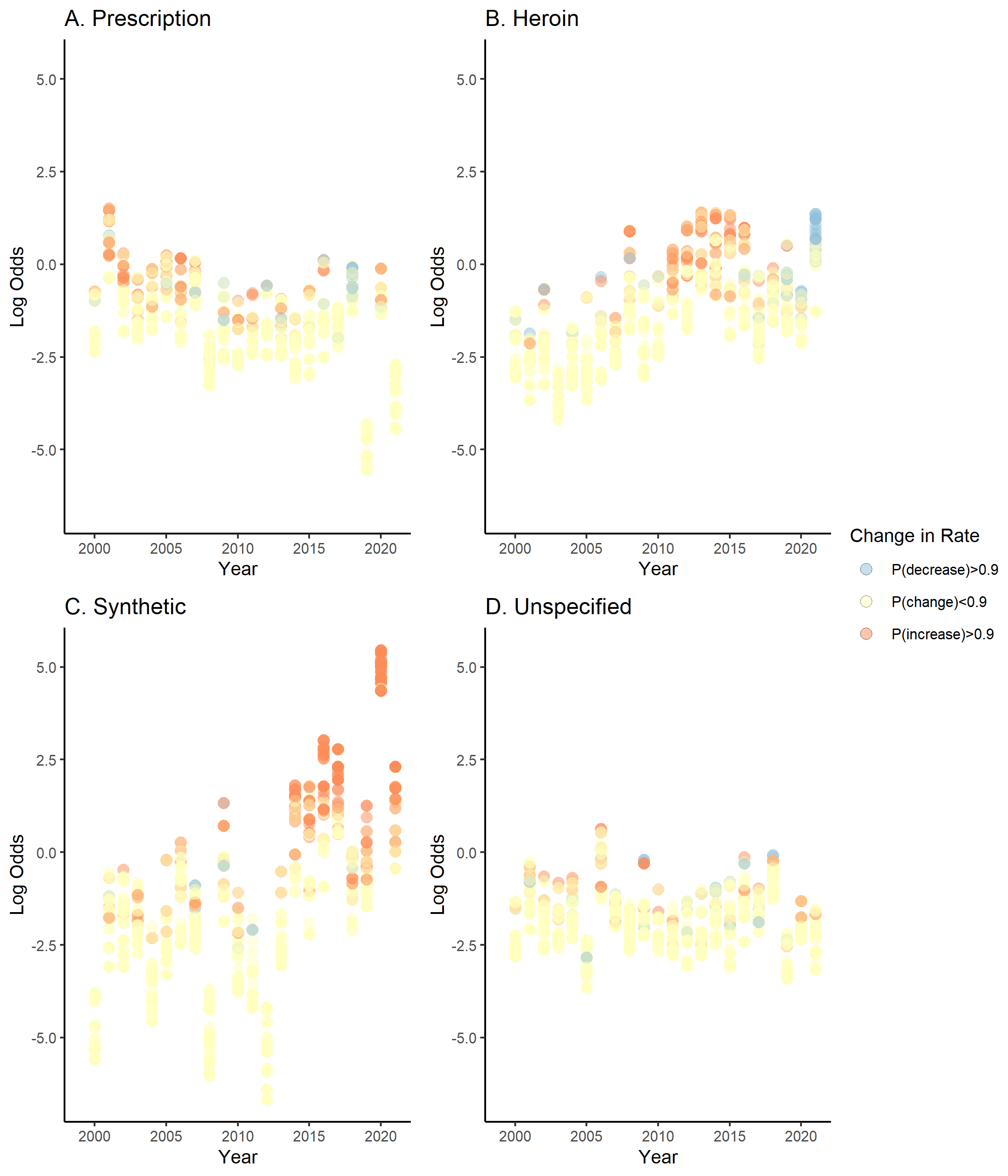

Supplement: S12 Fig — Posterior median estimates of the region-specific average log odds of a change in the mean overdose death rate for each state and drug type from 2000-2021. (TIF) [file pone.0309938.s015.tif]

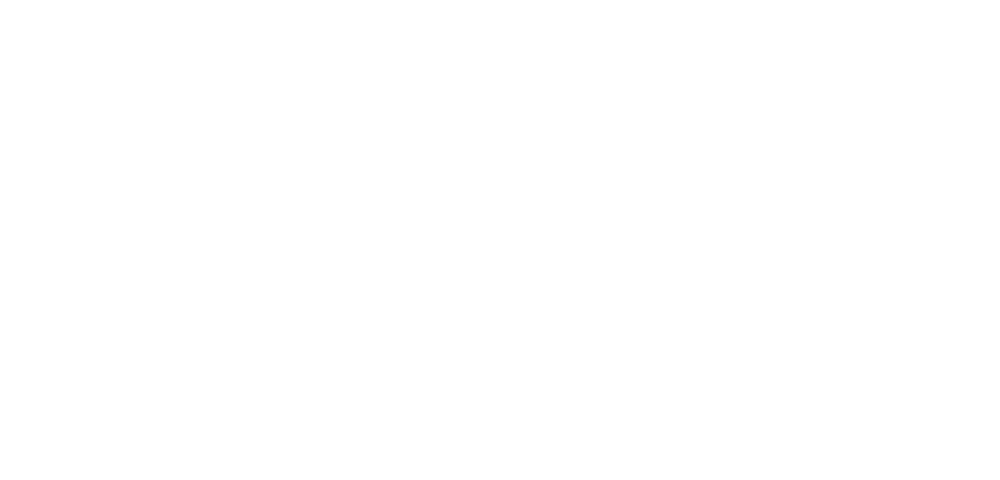

Supplement: S1 Video — Maps showing the posterior median overdose death rate per 100,000 residents for deaths involving each drug type from 1999-2021. Red outlines reflect a posterior probability of greater than 0.9 that the rate increased from the previous year, and blue outlines reflect a posterior probability of greater than 0.9 that the rates decreased from the previous year. (GIF) [file pone.0309938.s016.gif]

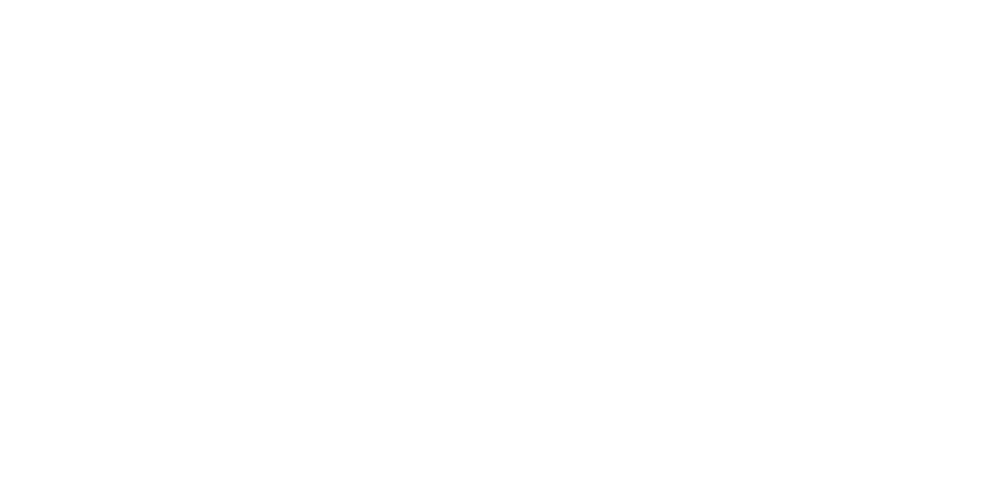

Supplement: S2 Video — Maps showing the posterior median log relative risk of death for the current year compared to the previous year for deaths involving each drug type from 2000-2021. Red outlines reflect a posterior probability of greater than 0.9 that the rate increased from the previous year, and blue outlines reflect a posterior probability of greater than 0.9 that the rates decreased from the previous year. (GIF) [file pone.0309938.s017.gif]
